# Quantitative analysis with volume rendering of pathological myopic eyes by high-resolution three-dimensional magnetic resonance imaging

**DOI:** 10.1097/MD.0000000000022685

**Published:** 2020-10-16

**Authors:** Jinqiong Zhou, Ying Tu, Qinghua Chen, Wenbin Wei

**Affiliations:** Beijing Tongren Eye Center, Beijing Key Laboratory of Intraocular Tumor Diagnosis and Treatment, Beijing Tongren Hospital, Capital Medical University, Beijing, China.

**Keywords:** magnetic resonance imaging, pathological myopia, regression model, vitreous, volume

## Abstract

Discovering a relationship between axial length and vitreous volume would be helpful since the axial length is easier to measure than magnetic resonance imaging (MRI) parameters. This study aimed to analyze the topography of human eyes with pathological myopia through volume rendering images by high-resolution 3D-MRI and to establish a model to estimate the vitreous volume.

This was a retrospective, non-randomized, controlled study of patients evaluated at Tongren Hospital from July 7, 2007 to December 12, 2018. The controls were emmetropic volunteers. All participants underwent ophthalmic examinations. Axial length was measured with an IOL Master. High-resolution 3D MRI and volume rendering was utilized for all the eyes. Logistic regression was used to establish a model to predict the vitreous volume.

A total of 280 emmetropic eyes and 290 eyes with pathological myopia were included. Males represented 60.7% and 65.5% of the individuals. The mean axial lengths of those two groups were 23.1 ± 0.8 mm (95%CI: 22.7–23.4 mm) and 28.3 ± 2.2 mm (95%CI: 27.5–29.2 mm), respectively (*P* < .001). The regression model in the pathological myopic group for calculating the vitreous volume according to the axial length was: Vitreous volume = 546.27 × axial length − 6977.12. The regression model in the emmetropic group for calculating the vitreous volume according to the axial length was: Vitreous volume = 458.35 × axial length − 6331.14 (*R*^2^ = 0.360, *P* = .001).

Elongation of the axial length is involved in eyeball enlargement in pathological myopic eyes. Measurement of the axial length could be recommended for the estimation of the vitreous volume during vitrectomy if vitreous cavity filling is needed.

## 1 Introduction

The prevalence of myopia varies widely around the globe, with 33% in the United States and 23% in Beijing, but can be as high as 90% in specific subpopulations such as students and young adults in East Asia.^[1]^ As a major cause of legal blindness worldwide, especially in East Asia,^[2–4]^ pathological myopia is characterized by high myopic refractive error and axial length elongation of the eye.^[5,6]^ Excessive elongation and deformity of the eyeball and posterior staphyloma are believed to be important factors in the development of the degenerative changes associated with pathological myopia, including chorioretinal atrophy, foveoschisis, choroidal neovascularization, retinal detachment, cataract, and glaucoma.^[7,8]^

Over the past two decades, advancement in imaging technologies have greatly enhanced our understanding of the ocular deformity associated with pathological myopia.^[9,10]^ It has proved that eyeball deformity in pathological myopia ranges from changes to the anterior chamber to the rear of the eyeball. Nevertheless, which part plays the most important role in the development of pathological myopia is still unknown. In addition, in clinical practice, knowledge of the vitreous volume is needed to determine the quantity of inert gas or silicone oil to be injected during vitrectomy, and the exact measurement of the circumference of the equator is needed during scleral buckling for rhegmatogenous retinal detachment. In addition, new therapeutic agents targeted the retina are increasingly being developed, injected or implanted into the vitreous cavity. Information regarding the vitreous volume would be important for us to understand the behavior of these agents once deposited into the vitreous. Recently, with the development of high-field magnetic resonance imaging (MRI) systems and newer designs of radiofrequency coils, the acquisition of volumetric ocular MRI data that have a high intrinsic resolution in all three-dimensions became feasible.^[11–13]^

Discovering a relationship between axial length and vitreous volume would be helpful in daily clinical practice since the axial length is easier to measure than MRI parameters. A study revealed that there are mathematical relationships between the axial length and vitreous volume, but that the relationship was different between macular hole and retinal detachment.^[14]^ Axial length can predict eye volume with an optimal *R*^2^ value of 79.4%.^[13]^ Nevertheless, data in myopic eyes are missing.

Therefore, the purpose of this study was to analyze the different sites of enlargement of pathological myopic eyes using 3D-MRI and to establish a correlation model to estimate the vitreous volume in pathological myopia.

## 2 Methods

### 2.1 Participants

This was a retrospective study that was conducted at the outpatient department of Ophthalmology of Tongren Hospital from July 2007 to December 2018. This study adhered to the tenets of the Declaration of Helsinki and was approved by the Ethics Committee of Beijing Tongren Hospital.

Patients with pathological myopia came from the outpatient department of Ophthalmology. The controls were emmetropic volunteers who were the current or former hospital staff members of Tongren Hospital and students of the Capital Medical University. The definition of pathological myopia was a spherical equivalent (SE) <−8.00 diopters (D) or axial length >26.5 mm, and emmetropia was defined as −1.0 D < SE < +1.0 D.

The inclusion criteria were:

1. met the diagnostic criteria of pathological myopia or emmetropia;
2. 18 to 60 years of age;
3. no history of corneal disease or other ocular diseases affecting diopter; and
4. no history of intraocular surgery or refractive surgery for myopia.

The exclusion criteria were:

1. uncontrolled severe heart, brain, lung, or kidney diseases;
2. pregnant or lactating women;
3. any contraindication to MRI; or
4. incomplete data.

This work has been carried out in accordance with the Declaration of Helsinki (2000) of the World Medical Association. This study was approved by the Medical Ethics Committee of Beijing Tongren Hospital. This article is a retrospective study. Therefore the Institutional waived the requirement to obtain distinct written informed consent from the patients.

### 2.2 Ophthalmic examinations

All participants underwent comprehensive ophthalmic examinations, including slit-lamp examination, measurement of the best-corrected visual acuity (BCVA), refractive error, axial length by the IOL Master system (Carl Zeiss Meditec, Jena, Germany), dilated fundus examination (three drops of tropicamide phenlyephine eye drops, given 5 min apart), and fundus photography (TRC-50DX; Topcon, Tokyo, Japan).

### 2.3 MRI scanning

The volumes of the lens, anterior chamber, vitreous, eyeball, and the axial length were measured by MRI. An MRI scanner (SignaHDxt 1.5T, version 15, GE Healthcare, Waukesha, WI) was used. The MRI scans were performed with an 8-channel phased-array head coil, which is able to rapidly scan both eyes simultaneously with a high signal-to-noise ratio. T2-weighted MRI, three-dimensional fast imaging employing steady-state acquisition (3D-FIESTA) was performed: 288 × 256 matrix, 1-mm slice thickness, repetition time (TR) of 63 ms, echo time (TE) of 221.606 ms, the spacing between slices: 0.4, reconstruction diameter: 140, and echo train length (ETL) of 54. The scan time for each subject was 2 min and 57 s. Volume renderings of the images were generated from high-resolution 3D data on a computer workstation (v. AW 4.4, GE Healthcare) to obtain high-resolution 3D data.

The margins of the eyeballs were detected semi-automatically by the signal intensity, and the tissues around the eyeballs were carefully removed. The boundary between the retina and vitreous fluid was determined by adjusting the signal intensity in the volume rendering of the MRI. When the signal intensity was gradually increased via manual mode, only the signal from vitreous fluid remained. De-noising, such as smoothing or curve/surface fitting, was done automatically by the computer workstation after volume rendering was done.

The area of each slice was measured semi-automatically on the computer workstation using the Image J software (National Institutes of Health, Bethesda, MD). The volume of each slice was calculated by area × slice thickness, and the total volume of each site was the sum of the volume of each slice. The largest slice of the lens, anterior chamber, and the vitreous were also obtained. The repeatability was confirmed by measuring the parameters of the 3D-MRI taken at different times by two experienced senior investigators (Ying Tu and Jinqiong Zhou, with more than 5 years of working experience). The investigators were not aware of grouping and diagnoses when measuring the MRI parameters.

To determine the inter- and intraobserver variability in the measurement of the volume of each slice, 100 random 3D MRI images were reviewed by the two investigators (Ying Tu and Jinqiong Zhou) independently in a masked manner. One observer (J.Q.Z.) re-examined the same 100 images again after 1 month.

### 2.4 Statistical analysis

Statistical analysis was performed using SPSS 20.0 for Windows (IBM, Armonk, NY). The continuous variables were tested for normal distribution using the Kolmogorov–Smirnov test and were found to follow the normal distribution. The continuous variables were expressed as means ± standard deviation (SD) and analyzed using the unpaired-samples *t* test with two tails. Categorical data were expressed as numbers and percentages and analyzed using the chi-square test. The correlation between axial length and vitreous volume, axial length and anterior chamber volume, axial length, and eyeball volume was analyzed by the Pearson correlation coefficient test. A logistic regression model was used to establish a model to predict the vitreous volume. *P* values <05 were considered statistically significant.

## 3 Results

### 3.1 Characteristics of the patients

A total of 323 consecutive participants (570 eyes) were enrolled in this study, including 153 participants with 280 emmetropic eyes, and 170 participants with 290 eyes with pathological myopia. The mean age of the participants with myopia and controls was 34.0 ± 11.9 years and 37.4 ± 14.3 years, respectively (*P* = .152). SE was smaller in the pathological myopia group compared with the emmetropic group (−9.57 ± 4.62 vs 0.05 ± 0.41, *P* < .001). There was no significant difference between groups regarding sex (male, 60.7% vs 65.5%, *P* = .551) (Table 1).

**Table 1:**
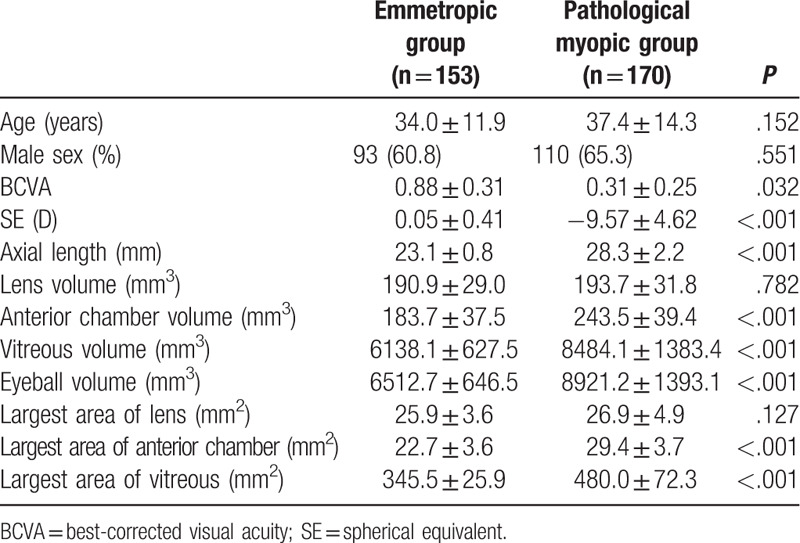
Demographic data and eye parameters of the participants.

### 3.2 MRI characteristics

The reproducibility of the assessments of the volume of each slice revealed kappa values of 0.89 for intraobserver variability and 0.97 for interobserver variability. In the emmetropic and myopic groups, the mean volume of the lens was 190.9 ± 29.0 vs 193.7 ± 31.8 mm^3^, respectively (*P* = .782). Compared with the emmetropic group, the mean volume of the anterior chamber (183.7 ± 37.5 vs 243.5 ± 39.4 mm^3^, *P* < .001) and the mean volume of the vitreous (6138.1 ± 627.5, 8484.1 ± 1383.4 mm^3^, *P* < .001) of the myopic group were larger. The eyeball volume showed the same trend (6512.7 ± 646.5 vs 8921.2 ± 1393.1 mm^3^*P* < .001) (Table 1).

As for the mean largest area of the lens, there was no significant difference between the two groups (25.9 ± 3.6 vs 26.9 ± 4.9 mm^2^, *P* = .127). Compared with the emmetropic group, the largest area of the anterior chamber (22.7 ± 3.6 vs 29.4 ± 3.7 mm^2^, *P* < .001) and the mean largest areas of the vitreous (345.5 ± 25.9 vs 480.0 ± 72.3 mm^2^, *P* < .001) of the myopic group were larger (Table 1).

### 3.3 Correlation between axial length and vitreous volume

**Figure 1:**
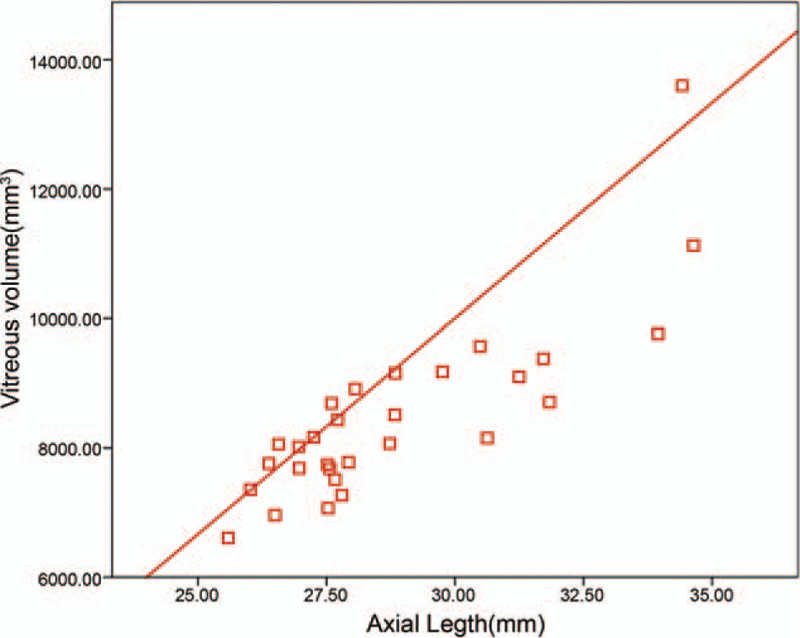
Pearson's correlation between axial length and vitreous volume in pathological myopia group.

**Figure 2:**
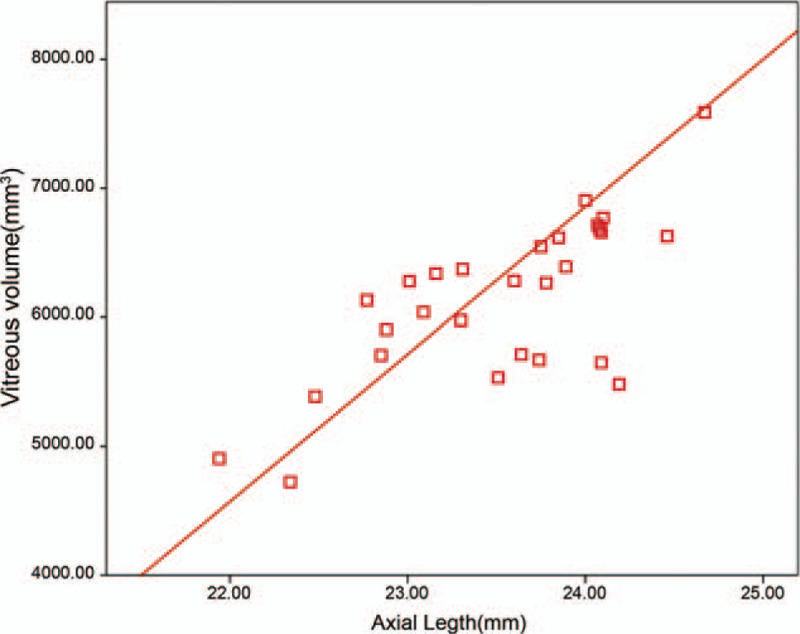
Pearson's correlation between axial length and vitreous volume in emmetropia group.

The mean axial lengths of those two groups were 23.1 ± 0.8 mm (95%CI: 22.7–23.4 mm) and 28.3 ± 2.2 mm (95%CI: 27.5–29.2 mm), respectively (*P* < .001). There was week correlation between axial length and anterior chamber volume (*r* = 0.48, *P* < .001), and strong correlation between axial length and eyeball volume (*r* = 0.91, *P* < .001). The mean vitreous volume was 4248.66 ± 358.12 mm^3^ for emmetropic group and 8482.32 ± 677.44 mm^3^ for pathological myopic group. The axial length and the vitreous volume were correlated in pathological myopic eyes (*r* = 0.84, *P* < .001) (Fig. 1) and emmetropic eyes (*r* = 0.71, *P* = .04) (Fig. 2). The regression model in the pathological myopic group for calculating the vitreous volume according to the axial length was: Vitreous volume = 546.27 × axial length − 6977.12 (*R*^2^ = 0.77, *P* < .001). The regression model in the emmetropic group for calculating the vitreous volume according to the axial length was: Vitreous volume = 458.35 × axial length − 6331.14 (*R*^2^ = 0.36, *P* = .001) (Table 2).

**Table 2:**
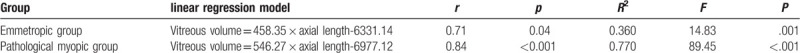
Correlation between axial length and the volume of vitreous.

## 4 Discussion

This study aimed to analyze the topography of human eyes with pathological myopia through volume rendering images by high-resolution 3D-MRI and to establish a correlation model to estimate the vitreous volume in pathological myopia. The results suggest that elongation of the axial length plays an important role in the enlargement of the eyeball in pathological myopic eyes. Measurement of the axial length could be recommended for the estimation of the vitreous volume during vitrectomy if vitreous cavity filling, therapeutic agents injection or implantation is needed.

This study showed that high-resolution 3D-MRI was an ideal choice for precise measurement of the ocular volume, with great reproducibility. High-resolution MRI with reconstructed 3D-MRI scans by volume rendering could help determine the ocular shape of myopia from any angle by rotating the image, with obvious advantages.^[7,15]^ Regarding the ultrasound, the frequency will determine the image resolution and resolution at higher depths, and the scattering of the sound beam by eye structures with different acoustic properties might lead to artifacts and decreased resolution. In addition, ultrasound is user-dependent.^[16]^ As for the Optical Coherence Tomography(OCT), even though it is superior in detecting details of iris-humor interface and corneal sublayer, the main disadvantage is that the light will be scattered by the structures of the eyes and that anterior chamber volume might be difficult to measure.^[16]^ Some researchers used the Swept-Source Optical Coherence Tomography (SS-OCT) to measure the anterior chamber volume of cataract patients, with a lower (139.80 ± 38.21 mm^3^) anterior chamber volume than our emmetropic group (183.7 ± 37.5 mm^3^).^[17]^ The reasons for the above-mentioned differences, except for the participants in their study(66.48 ± 10.57 years old) were much older than ours, the 3D resolution and accuracy of the OCT might need to be considered. Compare to the computed tomography (CT), MRI avoids health risks associated with ionising radiation found in routine X-rays and CT scans. Furthermore, the resolution is greater than that of traditional CT scanning. Azhdam et al^[18]^ reported a study about the measurement of the vitreous volume using Computed Tomography Imaging. The mean vitreous volume in their study was 4649.97 mm^3^, larger than emmetropic group (4248.66 ± 358.12 mm^3^) but small than pathological myopic group (8482.32 ± 677.44 mm^3^) in our study. Considering such difference, they did not differentiate the emmetropic eyes and myopic eyes. The mean axial length in their study was 24.7 ± 1.13 mm.^[18]^

This study showed that there was no significant difference in the volume of the lens between the emmetropic moderate myopic and pathological myopic groups. The volume of the anterior chamber of the pathological myopic group was larger than in the emmetropic group. The vitreous volume of the pathological myopic group was larger. The results were similar between the two groups when regarding the largest area of the lens, anterior chamber, and vitreous. And there was week correlation between axial length and anterior chamber volume, strong correlation between axial length and eyeball volume. These findings suggest that the enlargement of the anterior chamber and vitreous are features of pathological myopia, not only the longitudinal but also the horizontal extension. Wong et al^[19]^ reported that persons with a more negative spherical equivalents value had a deeper anterior chamber depth and longer axial length than subjects with a positive spherical equivalents value. Since the volume was calculated by area and thickness in the present study, we hypothesized that the depth of anterior chamber, especially the axial length, might play the most important role of the enlargement of the eyeball in pathological myopic eyes. In the present study, age and refractive diopter were not included in the regression model; only the axial length was included. This was because the axial length was a covariate with age and refractive diopter.^[20,21]^

For measuring axial lengths, we found out that IOL Master has several advantages over MRI and ultrasound, such as providing a true cornea-to-macula distance with patient fixation, avoiding interference from epiretinal membranes or foveoschisis because the measurement is done to the level of the retinal pigment epithelium.^[22]^ In this study, the mean axial lengths measured by an IOL Master of pathological myopic eyes were significantly larger than in emmetropic eyes. The relationship between vitreous volume and axial length can be presented as a regression model (vitreous volume = 546.27 × axial length − 6977.12) in the pathological myopic group. This model can be recommended for the estimation of the vitreous volume according to the axial length, which might be especially important when vitrectomy is performed, and vitreous cavity filling is needed. These results were supported by Nagra et al^[13]^ who also used the IOL Master and MRI to determine that the axial length can be used to estimate the eyeball volume. Tanaka et al^[14]^ reported wide variations in vitreous volumes between patients with macular hole and those with retinal detachment, but regression equations could be determined in each group between axial length and vitreous volume, based on direct gas volume injection during vitrectomy. This approach has the merit of providing the exact volume of gas being injected, but can only be performed in patients scheduled for surgery.

The potential limitations of our study should be considered. First, this was a hospital-based study, and the participants examined were volunteers in our hospital and the patients who visited our high myopia clinic. Thus, a referral bias cannot be ruled out principally, and the results may not represent the general myopic population. Second, the number of participants was still not large enough to determine how the enlargement of different sites contributed to the eyeball deformity in pathological myopic eyes. In addition, the axial length measured by IOL Master was the distance between cornea and macula. We are aware that the axial length was not the longest length in some eyes with eyeball deformity, which may lead to underestimation of the vitreous volume when calculating it according to the axial length.

## 5 Conclusion

This study showed that using high-resolution 3D-MRI and volume rendering, various deformities of the eyeball can be presented in most pathological myopic eyes, with the enlargement of the anterior chamber and vitreous. In pathological myopic eyes, the elongation of axial length played the most important role in the enlargement of eyeball. Axial length measurement could be recommended for the estimation of the vitreous volume during vitrectomy if vitreous cavity filling is needed.

## Author contributions

**Data curation:** Jinqiong Zhou, Qinghua Chen.

**Funding acquisition:** Wenbin Wei.

**Investigation:** Jinqiong Zhou, Ying Tu, Qinghua Chen.

**Methodology:** Ying Tu, Jinqiong Zhou.

**Project administration:** Ying Tu.

**Software:** Qinghua Chen.

**Validation:** Jinqiong Zhou.

**Writing – original draft:** Jinqiong Zhou, Wenbin Wei, Ying Tu.

**Writing – review & editing:** Jinqiong Zhou.

## References

[1] Wu PC Huang HM Yu HJ Epidemiology of Myopia. Asia Pac J Ophthalmol (Phila) 2016;5:386–93. 27898441 10.1097/APO.0000000000000236

[2] Dolgin E The myopia boom. Nature 2015;519:276–8. 25788077 10.1038/519276a

[3] Morgan IG French AN Ashby RS The epidemics of myopia: aetiology and prevention. Prog Retin Eye Res 2018;62:134–49. 28951126 10.1016/j.preteyeres.2017.09.004

[4] Morgan IG Ohno-Matsui K Saw SM Myopia. Lancet 2012;379:1739–48. 22559900 10.1016/S0140-6736(12)60272-4

[5] Wong TY Foster PJ Hee J Prevalence and risk factors for refractive errors in adult Chinese in Singapore. Invest Ophthalmol Vis Sci 2000;41:2486–94. 10937558

[6] Xu L Wang Y Li Y Causes of blindness and visual impairment in urban and rural areas in Beijing: the Beijing Eye Study. Ophthalmology 2006;113:1134.e1131–11. 16647133 10.1016/j.ophtha.2006.01.035

[7] Moriyama M Ohno-Matsui K Hayashi K Topographic analyses of shape of eyes with pathologic myopia by high-resolution three-dimensional magnetic resonance imaging. Ophthalmology 2011;118:1626–37. 21529960 10.1016/j.ophtha.2011.01.018

[8] Cho BJ Shin JY Yu HG Complications of pathologic myopia. Eye Contact Lens 2016;42:9–15. 26649982 10.1097/ICL.0000000000000223

[9] Curtin BJ The posterior staphyloma of pathologic myopia. Trans Am Ophthalmol Soc 1977;75:67–86. 613534 PMC1311542

[10] Ohno-Matsui K Lai T Lai C Updates of pathological myopia. Prog Retin Eye Res 2016;52:156–87. 26769165 10.1016/j.preteyeres.2015.12.001

[11] Tanitame K Sone T Miyoshi T Ocular volumetry using fast high-resolution MRI during visual fixation. AJNR Am J Neuroradiol 2013;34:870–6. 23042931 10.3174/ajnr.A3305 PMC7964486

[12] Velasco-Annis C Gholipour A Afacan O Normative biometrics for fetal ocular growth using volumetric MRI reconstruction. Prenat Diagn 2015;35:400–8. 25601041 10.1002/pd.4558 PMC4390455

[13] Nagra M Gilmartin B Logan NS Estimation of ocular volume from axial length. Br J Ophthalmol 2014;98:1697–701. 24985726 10.1136/bjophthalmol-2013-304652

[14] Tanaka H Nitoh K Atsuhiro A Measurement of volume of vitreous space during vitrectomy. Invest Ophthalmol Vis Sci 2009;13:3169.

[15] Moriyama M Ohno-Matsui K Modegi T Quantitative analyses of high-resolution 3D MR images of highly myopic eyes to determine their shapes. Invest Ophthalmol Vis Sci 2012;53:4510–8. 22678503 10.1167/iovs.12-9426

[16] Fujimoto JG Pitris C Boppart SA Optical coherence tomography: an emerging technology for biomedical imaging and optical biopsy. Neoplasia 2000;2:9–25. 10933065 10.1038/sj.neo.7900071 PMC1531864

[17] He W Zhu X Wolff D Evaluation of anterior chamber volume in cataract patients with swept-source optical coherence tomography. J Ophthalmol 2016;2016:8656301. 27688910 10.1155/2016/8656301 PMC5027314

[18] Azhdam AM Goldberg RA Ugradar S In vivo measurement of the human vitreous chamber volume using computed tomography imaging of 100 eyes. Transl Vis Sci Technol 2020;9:2. 10.1167/tvst.9.1.2 PMC7255624 32509437

[19] Wong TY Foster PJ Ng TP Variations in ocular biometry in an adult Chinese population in Singapore: the Tanjong Pagar Survey. Invest Ophthalmol Vis Sci 2001;42:73–80. 11133850

[20] Roy A Kar M Mandal D Variation of axial ocular dimensions with age, sex, height, BMI-and their relation to refractive status. J Clin Diagn Res 2015;9:AC01–4. 10.7860/JCDR/2015/10555.5445 PMC4347057 25737966

[21] Ip JM Huynh SC Kifley A Variation of the contribution from axial length and other oculometric parameters to refraction by age and ethnicity. Invest Ophthalmol Vis Sci 2007;48:4846–53. 17898312 10.1167/iovs.07-0101

[22] Wang NK Wu YM Wang JP Clinical characteristics of posterior staphylomas in myopic eyes with axial length shorter than 26.5 millimeters. Am J Ophthalmol 2016;162:180–90e181. 26585213 10.1016/j.ajo.2015.11.016

